# The Intention of Retail Stores in Taiwan to Cooperate with the Government in the Establishment of IT Measures for Pandemic Prevention

**DOI:** 10.3390/healthcare11010030

**Published:** 2022-12-22

**Authors:** I-Chiu Chang, Chih-Ming Chen, Wei-Chuan Lin, Der-Juinn Horng, Ying-Chin Ho, Hui-Ling Hu

**Affiliations:** 1 Department of Information Management, National Chung Cheng University, Chiayi 621301, Taiwan; 2 Department of Business Administration, National Central University, Taoyuan 320317, Taiwan; 3 Institute of Industrial Management, National Central University, Taoyuan 320317, Taiwan; 4 Quantitative Analysis and Research Association, Kaohsiung 800305, Taiwan

**Keywords:** COVID-19, perceived risk of infection, job stress, pandemic prevention IT measures, theory of planned behavior

## Abstract

This study focuses on the cooperative attitude and intention of retail stores in Taiwan to cooperate with the government’s related pandemic prevention measures. The study is based on the Theory of Planned Behavior (TPB). The study includes factors such as perceived risk of infection, job stress, pandemic prevention IT (information technology) convenience, pandemic prevention attitude, and pandemic prevention intention. Pandemic prevention attitude is used as a mediating variable to establish the research framework. This study collected research data through a questionnaire survey. A total of 457 valid questionnaires were collected through an electronic questionnaire platform. The findings showed that perceived risk of infection and pandemic prevention IT convenience had a positive and significant effect on pandemic prevention attitude (β = 0.567; β = 0.422) and pandemic prevention intention (β = 0.424; β = 0.296). Job stress has a significant negative effect on attitude (β = −0.173). In addition, job stress influenced intention through attitudes. Finally, perceived risk, job stress, and IT convenience had high explanatory power (R^2^ = 0.706) on attitudes. Perceived risk, IT convenience, and attitude also had moderate explanatory power (R^2^ = 0.588) on prevention intention. The study also suggests practical recommendations to improve and cooperate with pandemic prevention intention.

## 1. Introduction

At the end of 2019, outbreaks of the new coronavirus began around the world. The outbreak spread rapidly to Taiwan. The World Health Organization (WHO) also recognized and declared the COVID-19 outbreak as an international public health emergency in 2020. The WHO also declared the COVID-19 epidemic a global pandemic (Taiwan Ministry of Health and Welfare, Department of Disease Control). In the face of the fierce outbreak, the Taiwan government has been praised by the world for its preventive policies and advanced planning. Health experts from various countries have also proposed strategies and guidelines to prevent the rapid spread of the virus. For example, measured included providing a real-name registration information system, wearing masks, spraying alcohol, washing hands regularly, and avoiding from crowded places [1].

The SARS virus was introduced into Taiwan from abroad in March 2003. In April of the same year, an outbreak of infection occurred in Taipei City Heping Hospital. The government immediately closed the hospital. Seven health care workers lost their lives in the line of duty. In Taiwan, there were 346 confirmed cases and 73 deaths. Therefore, the panic in Taiwan reached an unprecedented level. At that time, the Taiwanese government did not have any policies and measures to prevent the pandemic, such as IT convenience. The Taiwan government has been able to learn from experience in combating large infectious diseases. In the face of the new pandemic, Taiwan has been able to effectively implement its pandemic prevention strategy. The main preventive measures are as follows. (i) Timely optimization of border quarantine and closure of the border during critical periods to break the transmission chain of the virus. (ii) Upgrade the medical system for infectious diseases, promote health education, and make it mandatory for all people to wear masks for pandemic prevention. (iii) Expand testing to track high-risk cases and strengthen home quarantine, etc. These strategies for pandemic prevention are based on the ability of government agencies to respond in a timely and cautious manner. The government has been able to provide information platforms such as IT for pandemic prevention and forward-looking deployment [2]. However, when the government first implemented these policies, the reactions of retail stores and the public were very different. Some denied the seriousness of the outbreak, while others rushed to buy masks. The retail stores reacted differently to the perception and severity of the outbreak.

Many scholars have investigated the impact of COVID-19 epidemic prevention issues and measured the impact of COVID-19 on human life and the environment from a public policy perspective [3], the structural impact of the spread of COVID-19 on the livelihood economy [4], and the government’s advocacy and approach to epidemic information [5]. However, the perceived risk of infection, job stress, and pandemic prevention IT convenience for retail stores have not been thoroughly explored.

During the pandemic prevention period in Taiwan, government agencies provided free pandemic prevention information technology systems for retail stores and the public to use. The information system included an online map of pandemic prevention masks, QRCode real-name registration, Taiwan Social Distance App, etc. The system was intended to enable service providers and the public to cooperate smoothly with the government’s pandemic prevention policies. The pandemic prevention information technology has contributed a lot to the government’s pandemic prevention measures. However, it does add a lot of work pressure and burden to retail stores. This study focuses on understanding the factors that influence the attitude and intention of retail stores in Taiwan to cooperate with government measures during the epidemic. Therefore, this study will examine the attitudes and intentions of retail stores in Taiwan to cooperate with the government’s epidemic prevention measures. Based on the Theory of Planned Behavior (TPB), the following five constructs are considered: perceived risk of infection, job stress, pandemic prevention IT convenience, pandemic prevention attitudes, and pandemic prevention intention. Pandemic prevention attitude was used as the mediating variable to establish the research framework. The study data were collected through a questionnaire survey and analyzed by structural equation modeling (SEM). This analysis included narrative statistical analysis, measurement model validation, and structural model validation to validate the research hypotheses and reach conclusions.

## 2. Background and Hypotheses Development

In early 2020, the New Coronavirus outbreak hit industries around the world. In particular, the service industry (including tourism, hotels, retail, restaurants, etc.) was been severely affected, and Taiwan’s service industry was not immune to the impact. Retail stores must maintain a high level of awareness and increase the perceived risk of infection at all times [6]. In this chapter, we will present the definition and meaning of the theory of planned behavior, the perceived risk of infection, job stress, and pandemic prevention IT convenience, as well as introduce related research, and derive research hypotheses.

### 2.1. Perceived Risk of Infection

In the current study, it was found that the COVID-19 pandemic situation affects public behavior. In particular, there is still uncertainty about the perceived risks associated with the infection and its consequences, causing panic in retail stores and consumers. Since COVID-19 is a sudden risk of infection, it is often discussed in television media or on the internet. Fear and anxiety about the virus can capture public attention and perception of the risk of infection [7]. Perceived risk of infectious disease combines emotional and cognitive components [8]. Therefore, the social phenomena that people experience and their consequences influence risk-related decision-making behavior [9]. For example, Yu et al. (2021) studied the effect of perceived risk on COVID-19 on consumers’ intention to return to hotels [10]. The study found that the perceived risk from COVID-19 had a significant negative impact on the intention to return to hotels. The perceived risk of infection is measured from two perspectives.

#### 2.1.1. Perceived Susceptibility

Perceived susceptibility is a reflection of an individual’s subjective risk of contracting a disease or finding themselves in a situation with a negative health outcome. Perceived susceptibility is important for routine health prevention behaviors. When people do not perceive themselves to be at risk for disease, they are less likely to take precautions that endanger not only themselves but also others [1]. The COVID-19 pandemic constitutes a complex psychological effect on the public worldwide. The susceptibility of the population to COVID-19 has been widely used in the preventive health behavior literature [6]. William noted that perceived susceptibility is associated with preventive behaviors for COVID-19. Perceived susceptibility is a major driver of the intention to prevent the disease [11]. Perceptual sensitivity has a key role in the prevention of infection [1,12,13]. Taken together, perceived sensitivity is one of the factors that influence the adoption of preventive measures for COVID-19. To explain these different responses, among the well-known models in psychology are the Health Belief Model and the Protective Motivation Theory. Both of these regulate the critical roles of an individual’s perceived sensitivity to health threats in the intention to engage in preventive health behaviors [1].

Given the importance of perceived sensitivity to routine health protective behaviors, health psychologists have shown the importance of this relationship in times of pandemics. The majority of participants considered COVID-19 to be a very serious disease. The perception of COVID-19 susceptibility varies among different groups. Mant et al. (2021) indicated that young people in general consider COVID-19 to be a serious disease. This study validates that young people support and accept government policies as a measure of protection and mitigation of susceptibility [11].

The COVID-19 pandemic has made perceived sensitivity a major topic of discussion. Gender, age, and marital status also influence the degree to which an individual perceives sensitivity. Empathy, self-efficacy, and imagination all influence the perception of sensitivity [14]. Therefore, this study concluded that the majority of workers in the service industry are young people in general. Therefore, the sensitivity to the risk of infection and the attitude and intention to cooperate with the government’s pandemic prevention policy are positive.

#### 2.1.2. Perceived Severity

Perceived severity is defined as the perceived association with potential or actual harm. Deng et al. (2020) examined the relationship between the perceived severity of COVID-19 and post-pandemic consumer intentions [15]. It was found that the high perceived severity of COVID-19 and the experience of living a monotonous life during the pandemic significantly increased the likelihood of post-pandemic consumption. This, in turn, significantly increased individuals’ tendency to make impulse purchases after the pandemic. The findings suggest that COVID-19 severity may influence people’s consumption patterns after a pandemic. Second, Prasetyo et al. (2020) found that the perceived severity of COVID-19 had a significant direct effect on consumer behavior, which led to the perceived effectiveness of preventive measures [16]. Fragkaki et al. (2021) showed that individuals with low perceived severity had the least behavioral change during COVID-19, while individuals with high perceived severity had the most behavioral change [17]. The study by Prasetyo (2020) also showed that perceived severity had a significant effect on the pandemic prevention intention. Individual pandemic prevention intention had a significant direct effect on actual prevention behaviors and adaptive behaviors [16].

In summary, this study concludes that retail stores’ perception of their own risk of infection is positive in terms of their attitude and intention to cooperate with the government’s pandemic prevention policy. Therefore, the following two hypotheses are proposed in this study:

**Hypothesis 1 (H1).**  *The risk of infection in retail stores had a positive and significant effect on their pandemic prevention intention*.

**Hypothesis 2 (H2).**  *The risk of infection in retail stores had a positive and significant effect on their pandemic prevention attitude*.

### 2.2. Job Stress

Job stress is defined as the phenomenon of physical and emotional reactions that occur in the workplace that are harmful to oneself [18]. Rangrez (2022) proposed that stressors and job insecurity positively affect job stress and eventually lead to the intention to resign from a job [19]. In the analysis of the study, it was shown that job stress had a significant effect on the intention to leave and job stress factors, including job insecurity. According to a 2018 study, job tension and job insecurity and the risk of disease transmission all contribute to increased job stress and intention to leave [20].

There are many studies related to job stress. For example, O’Brien (2021) indicated that during the COVID-19 pandemic, job stress was very high among hospital nursing staff. Moreover, job stress is inversely related to job quality [6]. Studies have also found that higher job stress reduces the ability of nursing staff to perform well [21]. The COVID-19 outbreak has intensified the psychological stress of health care workers. This includes prolonged exposure to COVID-19 patients, irregular working hours, and workload, which largely cause stress and fatigue among health care workers. These can affect the quality of work [22]. On the other hand, work stress and its dimensions including time pressure and anxiety are significantly related to the main dimensions of occupational fatigue [23]. Dual sources of stress for hotel workers during the COVID-19 pandemic, job insecurity, and risk of infection were identified as dual stressors. The panic caused by the pandemic accelerated the dual stressors. Both of these dual stressors lead to increased job stress and intention to leave the job [24].

In summary, this study concluded that during the COVID-19 pandemic, work stress would have a negative impact on work quality and work intentions. Relatively speaking, there is also a negative impact on the government’s requirement for retail stores to cooperate with related pandemic prevention measures. Therefore, this study concluded that the higher the perceived job stress of retail store employees, the more negative the attitude to cooperate with the government’s pandemic prevention measures. The following hypothesis is also proposed for this study:

**Hypothesis 3 (H3).**  *The job stress of retail store employees had a negative and significant effect on their attitude towards cooperation with the pandemic prevention measures*.

### 2.3. Pandemic Prevention IT Convenience

Pandemic prevention IT convenience refers to the convenience of using information technology (IT) to achieve a method of preventing the spread of a pandemic. Pandemic prevention IT includes pandemic prevention-related information systems, programs, functions, and equipment implemented by the government and is used in pandemic prevention management. The information platform or system is free for retail stores and the public to download and use. For example, stores can download the government’s QRCode real-name registration barcode, the online map of pandemic masks, the name-based rationing scheme for the Covid Rapid test, and other information systems. The Pandemic prevention IT convenience studied in this study is measured from two perspectives:

#### 2.3.1. IT Self-Efficacy

Self-efficacy refers to an individual’s judgment of his or her ability to organize and execute the required plan of action to achieve a given task [25]. Self-efficacy is a key construct of social cognitive theory, which refers to people’s beliefs about their ability to organize resources and execute the course of action needed to complete a task. Puozzo and Audrin, in a 2021 study, stated that self-efficacy refers to an individual’s perceptions and beliefs about their skills and that they are effectively mobilized to succeed in a given action. In addition, self-efficacy has been shown to have a significant impact on users’ learning effectiveness [26]. Therefore, IT self-efficacy refers to the degree of confidence in one’s IT abilities and the degree of certainty that one can perform IT-related tasks.

There are many studies related to IT self-efficacy. For example, Niu’s empirical study showed that people frequently use social software to share health information. Technology self-efficacy has a significant effect on social software use and health behavioral intentions. Other kinds of literature also suggest that social software use has a positive effect on health behaviors [27]. Furthermore, the Chamorro-Koc study in 2021 indicated that IT self-efficacy had a positive effect on the satisfaction of using wearable devices. The results of the study showed that past use and experience positively and significantly influenced people’s perceptions of IT self-efficacy. There is a positive significant effect on IT self-efficacy and trust in IT health technology when designing and evaluating IT health technology [28]. Further, Chandrasekaran’s study in 2022 indicated the key predictors of wearable device use among adults. Adults with technology self-efficacy are more likely to adopt and use wearable devices to track or monitor their health status. Therefore, the perception of self-efficacy is an important factor for users to use IT technology to promote self-health [29].

#### 2.3.2. Pandemic Prevention IT Ease of Use

Perceived ease of use is the degree to which a person believes that a system can be easily understood. The intensity of use and interaction between the user and the system can affect the ease of use [30]. Zhang et al. (2014) considers perceived ease of use of IT as the level of awareness of the user of the information system. Perceived ease of use is the ease of understanding and interacting with the information technology system [31].

There are many studies related to the ease of use of IT. For example, Sri used the Technology Acceptance Model (TAM) as a reference model to study the acceptance of e-learning platforms during the COVID-19 pandemic. The findings suggest that pandemic prevention IT convenience has a significant impact on perceived ease of use [32]. Next, Kusyanti investigated the effect of users’ intention to use smartphones and analyzed it using structural equation modeling (SEM). The results of the study showed that IT ease of use had a significant positive effect on users’ use of smartphones [33]. Furthermore, Ghosh and Jhamb investigated the impact of online virtual learning platforms on users’ learning needs during the COVID-19 pandemic. Users’ perceived ease of use had a positive and significant effect on satisfaction and behavioral intentions [34]. Finally, Basuki investigated the impact of people’s intention to watch videos on online platforms during the pandemic and their behavior. The study indicated that perceived ease of use positively influenced people’s intention and behavior to watch videos on online platforms [35].

In addition, Izuagbe used the Technology Acceptance Model (TAM) in combination with IT ease of use to examine users’ intention to accept technology. It was found that IT ease of use was positively significant to users’ intention to accept technology [36]. The TAM model was found to be effective in explaining the high perceived ease of use on users’ intention to accept and continue using the technology [37].

In summary, this study concludes that IT self-efficacy and ease of use of pandemic prevention IT form Pandemic prevention IT convenience. The higher the perception of retail stores on Pandemic prevention IT convenience, the higher their intention and attitude to cooperate with the governmental implementation of IT measure. Therefore, the following hypotheses are proposed in this study.

**Hypothesis 4 (H4).**  *Pandemic prevention IT convenience in retail stores has a positive and significant impact on pandemic prevention attitude*.

**Hypothesis 5 (H5).**  *Pandemic prevention IT convenience in retail stores has a positive and significant impact on pandemic prevention intention*.

### 2.4. Theory of Planned Behavior (TPB)

The theory of planned behavior used in this study is an adaptation of the Theory of Reasoned Action (TRA). The Theory of Reasoned Action is a conceptual model proposed by Fishbein and Ajzen in 1980. The model states that an individual’s behavior is influenced by his or her intention to act, and that intention to act depends on the individual’s attitudes and subjective norms toward the behavior. The theory of planned behavior is frequently applied in various areas of research. Broadly speaking, the theory is well supported by empirical research in the assessment of health behaviors. Attitudes, subjective norms, and perceived behavioral control of health behaviors can be used to predict different types of behavioral intentions with high accuracy. These behavioral intentions influence the actual behavior in terms of perceived behavioral control [38]. The theory of planned behavior suggests that an individual’s intention to engage in a particular behavior is the most important factor in assessing that behavior. Behavioral intention is determined by three important factors, namely, attitude, subjective norm, and perceived behavioral control. The theory of planned behavior suggests that individuals who maintain positive attitudes and strong perceived subjective norms when engaging in a particular behavior will develop a stronger intention to engage in that behavior [38]. Therefore, this study uses the theory of planned behavior to evaluate the attitude and intention of retail stores in Taiwan to cooperate with government-promoted pandemic prevention measures.

Social distance and business constraints in the face of the global COVID-19 pandemic increase people’s risk of perceived infection. In 2022, Mucinhato et al. [39] applied the Theory of Planned Behavior (TPB) and risk perception to home health management during the COVID-19 pandemic. The results of this study support the usefulness of the Theory of Planned Behavior (TPB) in consumer perceptions of the risk of infection. It also suggests that public health crises may lead to changes in consumer behavior related to infection risk. Next, some scholars have used TPB to explain the intent of adults to receive the COVID-19 vaccine. Positive attitudes toward vaccination, stronger subjective norms, and high cognitive behavioral control are currently associated with the intention to receive the vaccine [40]. Furthermore, Zhang used TPB as a framework for his study and concluded that parents with positive attitudes, strong subjective norms, and high perceived behavioral control had higher attitudes and intentions to have their children vaccinated. When parents believe that their children are at high risk for COVID-19 infection, there perceive the benefits to vaccinating their children to be more important than the side effects of the vaccine. Also, positive parental attitudes toward child vaccination and parental subjective norms of child vaccination encourage vaccine behavior. These findings have assisted government policymakers and public health practitioners in understanding the key messages for policy development to increase parents’ intention to vaccinate their children against COVID-19 [41].

Among the various Taiwan prevention measures, the government’s most high-profile measures are the various technology-integrated COVID-19 prevention policies. For example, the integration of government data allows health care workers to know the travel history of patients through their national health insurance cards. There are also aspects such as the linking of masks with the map of the pharmacy, the implementation of QRCODE real-name registration, and the current promotion of the social distance app in Taiwan, etc. Without the help of IT technology, it would take a lot of time and manpower to deal with the Pandemic prevention problems. This also highlights the fact that modern technology can provide more efficient solutions, saving these hidden costs and focusing on other more productive areas.

In this study, pandemic prevention attitude refers to the attitude of retail stores to cooperate in the prevention of the spread of the epidemic. The pandemic prevention intention refers to the intention to cooperate in the prevention of the spread of the epidemic. In summary, the more positive the attitude of retail stores to cooperate with prevention measures, the higher their intention to cooperate with prevention measures. Therefore, the following hypothesis is also proposed in this study.

**Hypothesis 6 (H6).**  *The attitude of retail stores to cooperate with preventive measures has a positive and significant effect on pandemic prevention intention with preventive measures*.

## 3. Materials and Methods

### 3.1. Research Framework

**Figure 1:**
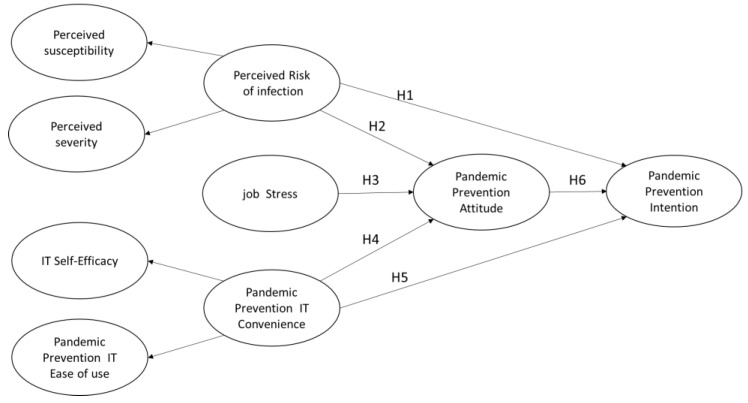
Research Framework.

In summary, this study suggests that perceived risk of infection, job stress, and pandemic prevention IT convenience have direct effects on attitudes and intentions to cooperate with pandemic prevention measures. For retail stores, the higher the perceived risk of infection, the stronger the attitude to cooperate, and the higher the pandemic prevention IT convenience, the more positive the attitude of retail stores to use. However, the higher the job stress, the lower the attitude of retail stores to cooperate with pandemic prevention. The higher the job stress, the more likely it is to cause a breach in pandemic prevention. This study predicts that the above hypothesis has a positive and significant effect on the attitude of retail stores to cooperate with epidemic prevention measures and their intention to cooperate with pandemic prevention measures. The research framework proposed in this study is as Figure 1.

### 3.2. Research Subjects and Data Collection

This study was conducted to investigate the intention of retail stores in Taiwan to cooperate with the government’s pandemic prevention IT and measures. Therefore, the target population of this study was retail stores in Taiwan. A questionnaire was used to conduct this study. A convenience sample survey was conducted by means of an online e-questionnaire. The questionnaires were distributed through online social groups and corporate email groups to collect data for the study, such as LINE groups, Facebook friends, IG communities, and email friends, etc. At the same time, a paper-based questionnaire survey was also conducted. An total of 210 questionnaires were completed by shopkeepers and sales clerks through a convenience sample of street stores conducted by work-study students. A total of 457 questionnaires were collected during the period of 1 June 2022 to 30 August 2022, and the validity rate was 100%.

#### 3.2.1. Personal Background Information

This study investigates the attitude and intention of retail stores in Taiwan to cooperate with the government’s pandemic prevention IT and measures during the COVID-19 pandemic prevention period. Therefore, basic personal information was measured in the questionnaire section. Four items were measured, including gender, age, education level, and occupation.

(1) Gender: There are two categories, male and female.

(2) Age: There are five categories, under 30, 31–40, 41–50, 51–60, and 61+.

(3) Educational level: There are four categories, junior high school, high school, college and university, and graduate school (or above).

(4) Occupation: There are six categories: food and beverage, retailers, tourism, transportation, leisure and entertainment, and others.

#### 3.2.2. Likert Seven-Point Scale

The questions in this study were measured using Likert’s seven-point scale. The range of scores was from strongly disagree (1), disagree (2), somewhat disagree (3), average (4), somewhat agree (5), agree (6), and strongly agree (7). The higher the score, the higher the level of agreement with the study variables. After the design of the questionnaire, expert scholars were invited to review the questions and give their opinions.

### 3.3. Description of the Research Variable

#### 3.3.1. Perceived Risk of Infection

Perceived risk of infection involves both susceptibilities to infection as well as perceived severity. The perceived susceptibility sub-construct of the perceived risk of infection in this study was based on Hsieh et al. This scholar proposed the Protection Motivation Theory (PMT) and the Unified Theory Technique of Acceptance and Use (UTAUT) model in 2017 [42]. In this study, the questions were modified according to the study context, and four questions were asked: “I think I have a high risk of infection with COVID-19”, “I would be worried about getting COVID-19”, “I think I am more likely to get COVID-19 than others”, and “I would be afraid of getting COVID-19”. Next, this study referred to the Health Belief Model (HBM) dimensions and risk-reducing behaviors on perceived severity as proposed by Li et al., 2019 [43]. The perceived severity questionnaire was modified according to the study context and five questions were derived: “I know that COVID-19 infection is a serious problem”, “I know that COVID-19 infection can lead to serious illness or even death”, “I feel that COVID-19 infection causes psychological fear”, “I feel that COVID-19 infection will increase the burden on my family”, “If I get COVID-19, my life will change”, and “I will be afraid of getting COVID-19”.

#### 3.3.2. Job Stress

This study refers to the study conducted by Lambert in 2018 [44] exploring the relationships between job stress, job engagement, and job satisfaction. This study used his questionnaire items, modified for this scenario, to obtain the job stress questionnaire as follows. “During pandemic prevention, things at job make me feel frustrated or angry”, “During pandemic prevention, I often feel stressed at work”, “During pandemic prevention, I often feel nervous”, “During pandemic prevention, my work often directly affects my health”, and “During pandemic prevention, I get irritable or nervous because of my work”. A total of five questions were designed.

#### 3.3.3. Pandemic Prevention IT Convenience

Pandemic prevention IT convenience includes the ease of use of prevention IT and the self-efficacy of prevention IT. This study is based on a study conducted by Kim et al. in 2010. The study was based on the empirical examination of the factors affecting cell phone usage intention, and the perceived ease of use showed a positive effect on user adoption intention [45]. Based on the study context, the questions of IT ease of use were modified and four questions were derived: “Pandemic prevention IT and measures are convenient at the business premises”, “Pandemic prevention IT and measures can be used by customers at any time at the business premises”, “Pandemic prevention IT and measures can be used by customers in any situation at the business premises”, and “Pandemic prevention IT and measures services at the business premises are not complicated at all”. This study draws on the studies by Yoo et al., 2018, exploring decision support systems for safety education [46] and Sánchez-Prieto et al. in 2017 to modify the questions of IT self-efficacy according to the research context [47], resulting in five questions. “I have the necessary skills to set up IT and measures for pandemic prevention in business premises”, “I have the necessary knowledge to set up IT and measures for pandemic prevention in business premises”, “I have the necessary ability to set up IT and measures for pandemic prevention in business premises”, “I know I can set up IT and measures for pandemic prevention in business premises even without being taught”, and “I can set up IT and measures for pandemic prevention in business premises even without being helped”.

#### 3.3.4. Pandemic Prevention Attitude

This study makes reference to Bae & Chang’s study in 2020 to study the effect of non-contact travel behavior intention during COVID-19 [13]. In addition, Hung-Chou et al., 2018, studied the effect of trust and attitude on the use of technology acceptance model integration among the elderly on the intention to reuse traceable agricultural products [48]. This paper was modified to fit the study context to derive four questions with the attitude of prevention. “I think it is useful to cooperate with government IT and measures for pandemic prevention”, “I think it is valuable to cooperate with government IT and measures for pandemic prevention”, “I think it is beneficial to cooperate with government IT and measures for pandemic prevention”, and “I am positive about using government IT and measures for pandemic prevention”.

#### 3.3.5. Pandemic Prevention Intention

This study refers to Shahid et al. study on the analysis of the human factors model affecting the intention to accept business intelligence (BI) [49]. In conjunction with and referencing Röttger et al., the study reveals the social perceptions of participation in workplace health promotion (WHP) using the theory of planned behavior [50]. The questions were modified according to the study context to match the questions on the intention to prevent IT and measures, resulting in five questions. “I will cooperate with government IT and measures for pandemic prevention”, “I will recommend to others to build government IT and measures for pandemic prevention”, “I am willing to participate in teaching or explaining government IT and measures for pandemic prevention”, “In principle, I have the desire to participate in government IT and measures for pandemic prevention”, and “In principle, I have the need to participate in government IT and measures for pandemic prevention”.

### 3.4. Data Analysis

In this study, SPSS 24.0 was applied to analyze the basic personal data, including the number and percentage of gender, age, education level, and occupation. AMOS 24.0 was used to analyze model convergent validity, discriminant validity, fit, path analysis, and mediation effects.

## 4. Research Results

### 4.1. Frequency Distribution Table

Service providers in Taiwan encompass a wide range of industries, including restaurants, retail stores, tourism, transportation, and leisure and entertainment. The 457 samples collected in this study were all valid samples and included in the study population. The majority of the respondents were male, 238 (52.1%). The average age of the respondents was below 30 years old, with 250 people accounting for 54.7% in this category. The average education level was below high school, with 157 people accounting for 34.4% in this category. The occupation was mainly in the field of retail trade, with 165 people accounting for 36.1% in this category, as Table 1 shown.

### 4.2. Convergent Validity

This study was analyzed according to the criteria suggested by Nunnally and Bernstein (1994) [51], as shown in Table 2. The standardized factor loadings ranged from 0.577–0.856, which were greater than 0.5, within a reasonable range, and showed that each question had question reliability. The reliability of the composition of the study constructs ranged from 0.833–0.921, all of which exceeded 0.7, indicating that the seven constructs were internally consistent. The final average variance extractions ranged from 0.528–0.700, all above 0.5, which also meets the criteria of Hair, Anderson, (1998) [52]. Fornell and Lacker’s (1981) [53] criterion indicates that the seven constructs in this study have good convergent validity.

### 4.3. Discriminant Validity

In this study, the more rigorous AVE method was used to examine the discriminant validity. According to Fornell and Lacker (1981) [53], discriminant validity should consider both convergent validity and construct correlation. Therefore, the square root of AVE for each construct must be larger than the correlation coefficient between the constructs. In this way, the study model can have discriminant validity. As shown in Table 3, the root-mean-square of AVE for each diagonal construct in this study is larger than the off-diagonal correlation coefficient, so this study has discriminant validity.

### 4.4. Model Fit Analysis

In this study, the Bollen–Stine Bootstrap was used to modify the model fit [54]. The comparison table of the results is shown in Table 4. After the Bollen–Stine Bootstrap modified model fit was calculated, all the fit indicators of this study passed and the results of this study were found to be acceptable.

### 4.5. Path Analysis

**Figure 2:**
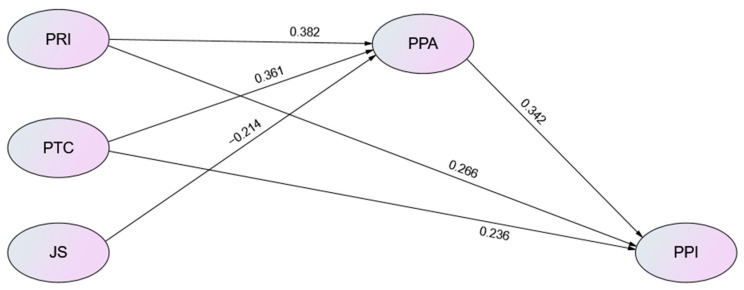
SEM Statistical Model of Path analysis results. Note: PPA = Pandemic Prevention Attitude; PPI = Pandemic Prevention Intention.; PRI = Perceived Risk of Infection; JS = Job Stress; PTC = Pandemic Prevention IT Convenience.

From Table 5 and Figure 2, the results of the path analysis are as follows. Perceived Risk of infection (b = 0.567, *p* < 0.05), Job Stress (b = −0.173, *p* < 0.05), and Pandemic prevention IT (b = 0.422, *p* < 0.05) significantly influenced Pandemic Prevention Attitude.

Pandemic Prevention Attitude (b = 0.367, *p* < 0.05), Perceived Risk of infection (b = 0.424, *p* < 0.05), and Pandemic prevention IT Convenience (b = 0.296, *p* < 0.05) significantly influenced Pandemic Prevention Intention. Perceived Risk of infection, Job Stress, and Pandemic prevention IT can explain 70.6% of Pandemic Prevention attitudes. Pandemic Prevention Attitude, Perceived Risk of infection, and Pandemic prevention IT Convenience can explain 58.8% of Pandemic Prevention Intention.

### 4.6. Mediating Effect

From Table 6, job stress → pandemic prevention attitude → pandemic prevention intention, *p* < 0.05, the confidence interval did not contain 0 [−0.189 −0.005], indicating that the indirect effect was valid.

## 5. Conclusions and Discussion

During the pandemic prevention period in Taiwan, the government provided free pandemic prevention information technology systems for retail stores and the public to use. The information system includes an online map of pandemic prevention masks, QRCode real-name registration, and a Taiwan social distance app. The pandemic prevention information technology has indeed added a lot of credit to the government’s pandemic prevention measures in an attempt to enable retail stores and the public to cooperate with the government’s pandemic prevention policies. However, it does add a lot of work pressure and burden to retail stores. This study was conducted to understand the factors influencing the attitude and intention of retail stores in Taiwan to cooperate with government measures during the outbreak. Therefore, this study was conducted to examine the attitudes and intentions of retail stores in Taiwan when cooperating with the government’s pandemic prevention measures. Among the 457 samples collected in this study, the ratio of male to female was approximately the same. The majority of the samples were under 30 years old and 31–40 years old. The education level was mostly high school and junior college. Retail stores were mostly retailers, food and beverage, and tourism. It is evident that the interviewees do not have special competence and expertise in the use of IT for vaccination. Although most of the people who use IT are young, IT for epidemic prevention is a technology that has never been used before and is not an application that the average person would have access to.

Based on the theory of planning behavior, the five constructs of Perceived Risk of infection, job stress, pandemic prevention IT convenience, pandemic prevention attitude, and pandemic prevention intention were considered. The research framework was established by using pandemic prevention intention as the mediating variable. The study data were collected through a questionnaire survey and analyzed using structural equation modeling (SEM). This included narrative statistical analysis, measurement model validation, and structural model validation to validate the research hypotheses and draw conclusions from the study.

### 5.1. Discussion

This study is an application of a theory of planned behavior model to the recommendations and improvements of governmental pandemic prevention measures during the COVID-19 outbreak. The main contributions displaced are as follows:

- The perceived risk of infection and pandemic prevention IT convenience positively and significantly influenced the attitude and intention to cooperate with pandemic prevention measures. The effect of the perceived risk of infection was the most significant, and the results of this study were similar to those of Venema et al. [1]. In 2021, service industry workers in Taiwan have the greatest concern and influence on the perceived risk of infection. In addition, pandemic prevention IT convenience also positively and significantly influenced the attitude and intention of service industry workers to cooperate with pandemic prevention measures. The results of the study are similar to those of the study by Basuki et al. [35] in 2022. It is evident that pandemic prevention IT convenience has significant importance in outbreak prevention measures.
- Job stress in retail stores had a negative and significant effect on the attitude towards cooperation with pandemic prevention measures, similar to the findings of the study by Etesam et al. [23] in 2021. The more stressful it is to work during an outbreak, the less cooperative people will be to the government’s preventive measures. This increases the likelihood of causing a breach in the pandemic prevention. Furthermore, attitudes toward preventive measures positively and significantly influenced intention to cooperate with preventive measures, similar to Ahmad et al.’s [55] study in 2020, further validating the direct effect of attitudes on intention in the theory of planned behavior.
- The mediating effect of pandemic prevention attitude on the perceived risk of infection, job stress, and pandemic prevention IT convenience and pandemic prevention intention. It is evident that pandemic prevention attitude directly affects pandemic prevention intention.

### 5.2. Conclusions

This study confirms the importance of the perceived risk of infection and pandemic prevention IT convenience on attitude and intention to cooperate with pandemic prevention measures. Therefore, to improve the attitude and intention of retail stores to cooperate with pandemic prevention measures, it is necessary to improve the perceived risk of infection and pandemic prevention IT convenience of retail stores. The recommendations of this study for the government and the industry to prevent the epidemic are as follows.

- Raise awareness of the risk of the disease among service providers:

The government can publicize the severity of COVID-19 disease, its consequences, and the risk of re-infection through the media from different levels and perspectives of experts and academics. Owners should inform their staff and educate their first-line staff to raise their awareness of disease prevention.

- Enhance the convenience of pandemic prevention IT:

Government-provided or developed IT information systems should take into account the convenience of people’s use. This includes easy to use, easy-to-download, clear, and easy-to-understand interfaces, etc., to enhance the convenience of using IT for pandemic prevention.

- Enhance the safety protection of service staff:

The work protection measures for retail stores during the epidemic prevention period should be considered thoroughly. Measures should reduce the risk of epidemic prevention, reduce work pressure, and make employees feel safe in the working environment.

### 5.3. Research Limitations and Future Developments

This study was only conducted in retail stores. The study population was limited to workers in retail stores. The structure of the study focused on the perceived risk of infection, job stress, pandemic prevention IT convenience, attitude, and intention to cooperate with pandemic prevention measures. A sample of 457 respondents was collected from retail stores in Taiwan for analysis. The advantage of using social media to collect data is that the retailers can help to recommend their peers to fill in the data and collect the questionnaire data more efficiently. However, the disadvantage is that the data may be more homogeneous. Future research suggests that the target population can be expanded. In addition to retail stores, manufacturing and education industries should also be considered. Secondly, other research constructs, such as motivation and habits of pandemic prevention, can be added to the study. Further study should investigate the factors influencing the public’s preventive measures or behavioral changes under the influence of COVID-19.

## Figures and Tables

**Table 1:** Frequency distribution table.

Category	Group	Frequency	Percentage
Gender	Male	238	52.1
	Female	219	47.9
Age	Under 30	250	54.7
	31–40	104	22.8
	41–50	68	14.9
	Over 51	35	7.7
Education level	Below high school	157	34.4
	Junior college	147	32.2
	University	123	26.9
	Graduate school and above	30	6.6
Occupation	Food & Beverage	122	26.7
	Retailers	165	36.1
	Tourism	105	23.0
	Transportation	43	9.4
	Leisure and entertainment	13	2.8
	Other	9	2.0

**Table 2:** Convergent validity.

Construct	Item	Item Reliability		Construct Reliability	Convergence Validity
		Std.	SMC	CR	AVE
Perceived susceptibility	PSU1	0.619	0.383	0.820	0.534
	PSU2	0.754	0.569		
	PSU3	0.734	0.539		
	PSU4	0.804	0.646		
Perceived severity	PSE1	0.781	0.610	0.848	0.529
	PSE2	0.773	0.598		
	PSE3	0.742	0.551		
	PSE4	0.745	0.555		
	PSE5	0.577	0.333		
Pandemic Prevention IT Ease of use	ITE1	0.737	0.543	0.842	0.572
	ITE2	0.758	0.575		
	ITE3	0.780	0.608		
	ITE4	0.750	0.563		
IT Self Efficacy	ITS1	0.697	0.486	0.847	0.528
	ITS2	0.781	0.610		
	ITS3	0.797	0.635		
	ITS4	0.727	0.529		
	ITS5	0.615	0.378		
Job Stress	JS1	0.842	0.709	0.904	0.654
	JS2	0.826	0.682		
	JS3	0.788	0.621		
	JS4	0.787	0.619		
	JS5	0.800	0.640		
Pandemic Prevention Attitude	AT1	0.745	0.555	0.835	0.559
	AT2	0.751	0.564		
	AT3	0.775	0.601		
	AT4	0.718	0.516		
Pandemic Prevention Intention	IN1	0.768	0.590	0.883	0.602
	IN2	0.783	0.613		
	IN3	0.765	0.585		
	IN4	0.785	0.616		
	IN5	0.777	0.604		

Note: Std. = Standardized factor loading; SMC = Squared multiple correlation; CR = Composite reliability; AVE = Average variance extracted.

**Table 3:** Discriminant validity.

Construct	AVE	PSC	PS	PTU	ISE	JS	PPA	PPI
PSC	0.534 *	**0.737**						
PS	0.529 *	0.616 *	**0.727**					
PTU	0.572 *	0.401 *	0.440 *	**0.756**				
ISE	0.528 *	0.525 *	0.403 *	0.693 *	**0.727**			
JS	0.654 *	−0.515 *	−0.518 *	−0.484 *	−0.504 *	**0.809**		
PPA	0.559 *	0.618 *	0.584 *	0.659 *	0.579 *	−0.678 *	**0.748**	
PPI	0.602 *	0.560 *	0.529 *	0.563 *	0.558 *	−0.521 *	0.725 *	**0.776**

Note1: AVE = Average Variance Extraction; PSC = Perceived Susceptibility; PS = Perceived severity; PTU = Pandemic Prevention IT Ease of use; ISE = IT Self Efficacy; JS = Job Stress; PPA = Pandemic Prevention Attitude; PPI = Pandemic Prevention Intention. Note2: * *p* < 0.05.

**Table 4:** Goodness of fit index.

Fit Indices	Criteria	Result	Supported or Not
Chi-square		570.511	
Degree of freedom		451	
CFI	>0.9	0.981	Supported
RMSEA	<0.08	0.024	Supported
TLI	>0.9	0.988	Supported
GFI	>0.9	0.951	Supported
NFI	>0.9	0.951	Supported
χ2/df	<3	1.265	Supported
AGFI	>0.9	0.941	Supported

**Table 5:** Research hypothesis results.

	IV	Unstd.	S.E.	CR	*p*-Value	Std.	R^2^
PPA	PRI	0.567	0.137	4.151	0.000	0.382	0.706
	JS	−0.173	0.050	−3.454	0.000	−0.214	
	PTC	0.422	0.091	4.622	0.000	0.361	
PPI	PPA	0.367	0.106	3.466	0.000	0.342	0.588
	PRI	0.424	0.152	2.783	0.005	0.266	
	PTC	0.296	0.105	2.812	0.005	0.236	

Note: IV = Independent Variable; PPA = Pandemic Prevention Attitude; PPI = Pandemic Prevention Intention.; PRI = Perceived Risk of Infection; JS = Job Stress; PTC = Pandemic Prevention IT Convenience.

**Table 6:** The analysis of indirect effects.

Parameter	Estimate	Lower	Upper	*p*
Job stress → Pandemic Prevention Attitude → Pandemic Prevention Intention	−0.064	−0.189	−0.005	0.033

## Data Availability

Data sharing not applicable.
